# “Integrative genomic analysis of the bioprospection of regulators and accessory enzymes associated with cellulose degradation in a filamentous fungus (*Trichoderma harzianum*)”

**DOI:** 10.1186/s12864-020-07158-w

**Published:** 2020-11-02

**Authors:** Jaire A. Ferreira Filho, Maria Augusta C. Horta, Clelton A. dos Santos, Deborah A. Almeida, Natália F. Murad, Juliano S. Mendes, Danilo A. Sforça, Claudio Benício C. Silva, Aline Crucello, Anete P. de Souza

**Affiliations:** 1 Center for Molecular Biology and Genetic Engineering (CBMEG), University of Campinas (UNICAMP), Campinas, SP Brazil; 2 Graduate Program in Genetics and Molecular Biology, Institute of Biology, UNICAMP, Campinas, SP Brazil; 3 Holzforshung München, TUM School of Life Sciences Weihenstephan, Technical University of Munich, Freising, Germany; 4 Department of Plant Biology, Institute of Biology, UNICAMP, Campinas, SP Brazil; 5 Dept. de Biologia Vegetal, Universidade Estadual de Campinas, Campinas, São Paulo CEP 13083-875 Brazil

**Keywords:** Cellulose degradation, CAZymes, Genomic, Transcriptome, Fungi

## Abstract

**Background:**

Unveiling fungal genome structure and function reveals the potential biotechnological use of fungi. *Trichoderma harzianum* is a powerful CAZyme-producing fungus. We studied the genomic regions in *T. harzianum* IOC3844 containing CAZyme genes, transcription factors and transporters.

**Results:**

We used bioinformatics tools to mine the *T. harzianum* genome for potential genomics, transcriptomics, and exoproteomics data and coexpression networks. The DNA was sequenced by PacBio SMRT technology for multiomics data analysis and integration. In total, 1676 genes were annotated in the genomic regions analyzed; 222 were identified as CAZymes in *T. harzianum* IOC3844. When comparing transcriptome data under cellulose or glucose conditions, 114 genes were differentially expressed in cellulose, with 51 being CAZymes. CLR2, a transcription factor physically and phylogenetically conserved in *Trichoderma* spp., was differentially expressed under cellulose conditions. The genes induced/repressed under cellulose conditions included those important for plant biomass degradation, including CIP2 of the CE15 family and a copper-dependent LPMO of the AA9 family.

**Conclusions:**

Our results provide new insights into the relationship between genomic organization and hydrolytic enzyme expression and regulation in *T. harzianum* IOC3844. Our results can improve plant biomass degradation, which is fundamental for developing more efficient strains and/or enzymatic cocktails to produce hydrolytic enzymes.

**Supplementary information:**

**Supplementary information** accompanies this paper at 10.1186/s12864-020-07158-w.

## Background

*Trichoderma* is a very diverse genus of fungi that produces enzymes applied in different areas; some strains are applied in biocontrol (*T. atroviride*, *T. harzianum* and *T. virens*) [1, 2], and others are specific for the biofuel technology (*T. reesei* and *T. harzianum*) [3, 4]. Different strains of *T. harzianum* have high cellulolytic activity, and the potential of these enzymes has been explored for applications in biomass degradation to the production of biofuels [4, 5].

*Trichoderma harzianum* is a common fungal species in soil and is used as a biological control against a variety of phytopathogenic fungi [6]. Despite that, *T. harzianum* capacity to biomass degradation is still poorly explored compared to that of other cellulolytic fungi. Due to the high cellulolytic activity some strains has shown considerable potential for application in plant biomass hydrolysis [4, 7, 8]. *T. harzianum* strains have potential for the production of an enzymatic/protein arsenal necessary for the complete hydrolysis of cellulosic compounds in fermentable sugars [5, 9–11].

Currently, the most-studied and widely used industrial-scale enzymes are produced by the fungus *T. reesei* and species from the *Aspergillus* genus. These organisms are the sources of most enzymes comprising enzymatic cocktails that are currently available on the market [12]. *T. reesei* is a widely studied fungus and is found in several works on genomics, transcriptomics, proteomics and metabolic engineering [3, 13–16]. Thus, increasing the number of studies related to the biotechnological potential of *T. harzianum* is necessary.

Different strains of a specific fungal species have different potentials for the degradation of plant biomass, and these differences may be associated with differences in the genome and regulation of CAZyme enzymes [17]. *T. harzianum* IOC3844 is a strain that showed high potential for the degradation of plant biomass in several studies [4, 10], demonstrated via the high expression of genes related to cellulose (CEL) and hemicellulose degradation [18], enzymatic activity [4, 17] and synthetic biology [19].

The three main groups involved in the hydrolysis of CEL are cellobiohydrolases, endo-β-1,4-glucanases and β-glucosidases. In addition, accessory enzymes such as copper-dependent lytic polysaccharide mono-oxygenases (LPMOs), cellulose-induced protein (CIP1 and CIP2) and swollenin also participate in this process [20–23].

One of the great challenges in understanding the molecular mechanism of biomass degradation is to capture how the transcription factors (TFs) act. Several fungal TFs have been related to the degradation of plant biomass, many of which belong to the binuclear zinc family [24]. Many TFs have been described as directly involved in the regulation of plant biomass [25]. This number has expanded rapidly in recent years, mainly due to the development of whole genome sequencing technologies associated with the exponential increase in the number of bioinformatics analysis tools that produced massive amounts of information and increased the numbers of identified genes [25, 26].

The present study aimed to analyze genomic regions with CAZyme genes using a bacterial artificial chromosome (BAC) library built in house [27] and to integrate these data with RNA-Seq, secretome data and coregulation networks. We sequenced a massive amount of DNA and used the information obtained to integrate genomic data (genomic regions containing CAZymes), expression patterns (transcriptome under degradation conditions), proteins (secretome by mass spectrometry) and systems biology (with gene regulatory networks) to obtain a broad and precise overview of the CEL degradation pathways. Our study characterized the main genes, accessory enzymes and regions involved in the degradation and regulation of hydrolytic enzymes. In addition, we analyzed the regulator cellulose degradation regulator 2 (CLR2) found in a cluster with other important enzymes. These results will be important for further studies on regulation and gene silencing.

## Results

### Genomic regions of T. harzianum IOC3844

In this study, a library of large genomic regions was used as a platform to search for genes of interest and to thoroughly study the genomic structure of *T. harzianum* IOC3844 (ThIOC3844) (Additional file 1: Fig. S1 and Additional file 2: Supplementary Table S1). Screening for genes of interest resulted in a total of 62 regions that contained CAZyme genes related to the degradation of plant biomass in the ThIOC3844 genome. Sequencing of these regions generated 5 Mb total of the estimated 40 Mb genome (Additional file 1: Supplementary Table S2 and S3). These regions ranged in size from 43 to 152 kb, enabling the prediction and annotation of 1676 gene models for this strain (Additional file 3: Supplementary Table S4). The average number of genes per region was 26 (Additional file 2: Supplementary Table S1).

**Fig. 1:**
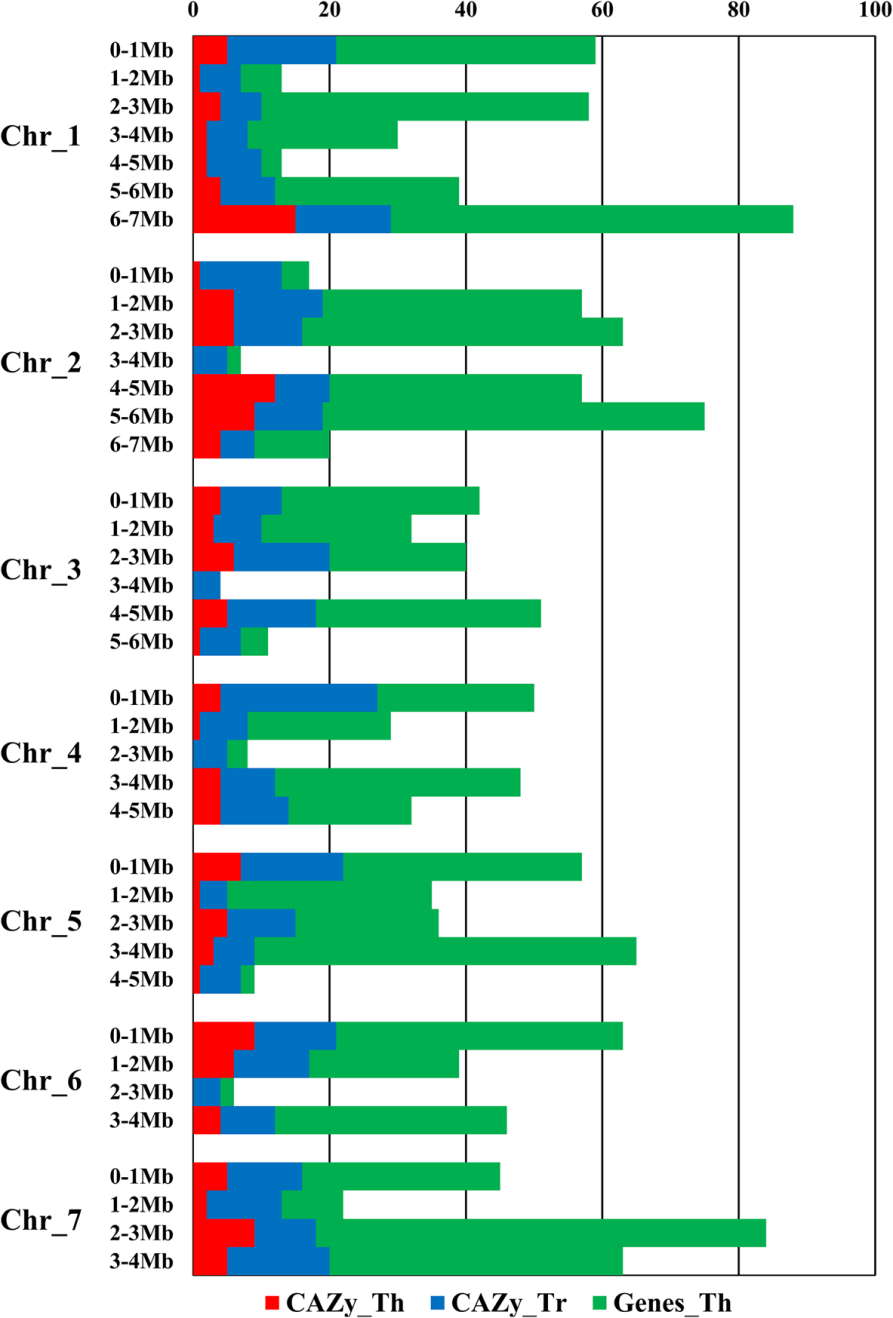
Distribution of the *T. harzianum* IOC3844 genes on the 1 Mb intervals of the seven chromosomes of *T. reesei* QM6a. CAZyme genes of *T. harzianum* IOC3844 are in red, CAZyme genes of *T. reesei* are in blue, and all genes of *T. harzianum* IOC3844 are in green. Th: *T. harzianum* IOC3844; Tr: *T. reesei* QM6a

The genome of *T. reesei* QM6a (PRJNA325840) was used to analyze the distribution of genes in ThIOC3844. This genome, composed of seven chromosomes with a total size of 34 Mb, was divided into 38 intervals (1 Mb) (Fig. 1). CAZyme genes annotated in ThIOC3844 were distributed throughout all *T. reesei* QM6a (TrQM6a) chromosomes. It was possible to map ThIOC3844 genes at all intervals in which the chromosomes were divided; mapping CAZyme genes was not possible in only four intervals.

**Fig. 2:**
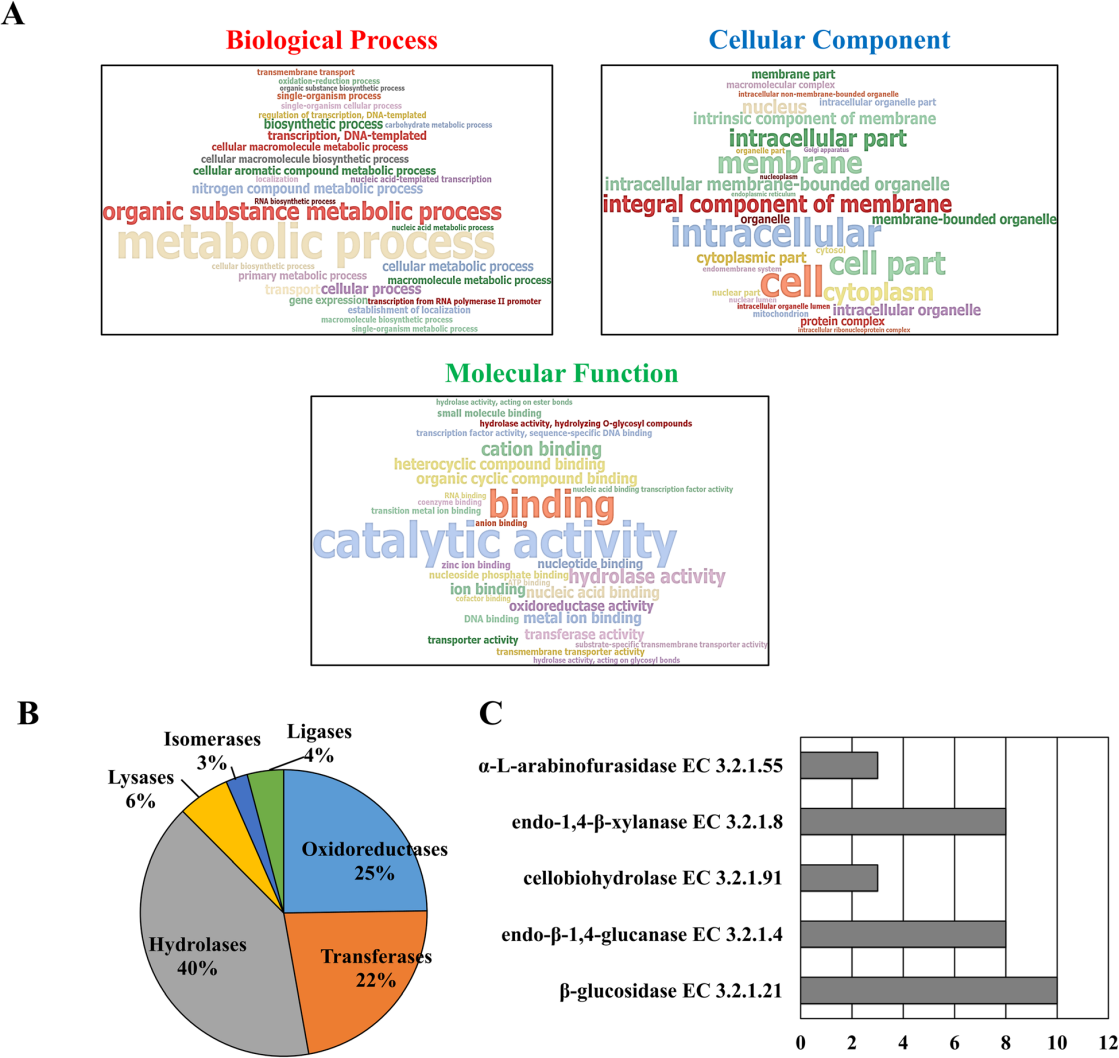
Functional annotation of the genes predicted in the genomic regions of *T. harzianum* IOC3844. Annotation of genes for ontologies of biological processes, cellular components and molecular functions. **a** Distribution of enzymes annotated according to enzyme commission (**b**) and major enzyme commission (EC) related to cellulose and hemicellulose degradation (**c**)

The genes were functionally annotated for the main gene ontologies: biological processes, cellular components and molecular functions (Fig. 2a and Additional file 1: Supplementary Fig. S2). We found 209 sequences related to hydrolytic activity, 139 related to transport proteins and 85 sequences related to the regulation of gene expression (possible TFs). In addition, a specific annotation was made for genes identified as enzymes, among which hydrolases (40%), oxidoreductases (25%), transferases (22%), lyases (6%), ligases (4%) and isomerases (3%) (Fig. 2b) were found. We also identified genes directly related to the degradation of CEL and hemicellulose, with activities of α-L-arabinofuranosidase (enzyme commission (EC) 3.2.1.55), endo-1,4-β-xylanases (EC 3.2.1.8), cellobiohydrolases (3.2.1.91), endo-β-1,4-glucanase (EC 3.2.1.4) and β-glucosidase (EC 3.2.1.21) (Fig. 2c and Additional file 4: Supplementary Table S5).

**Fig. 3:**
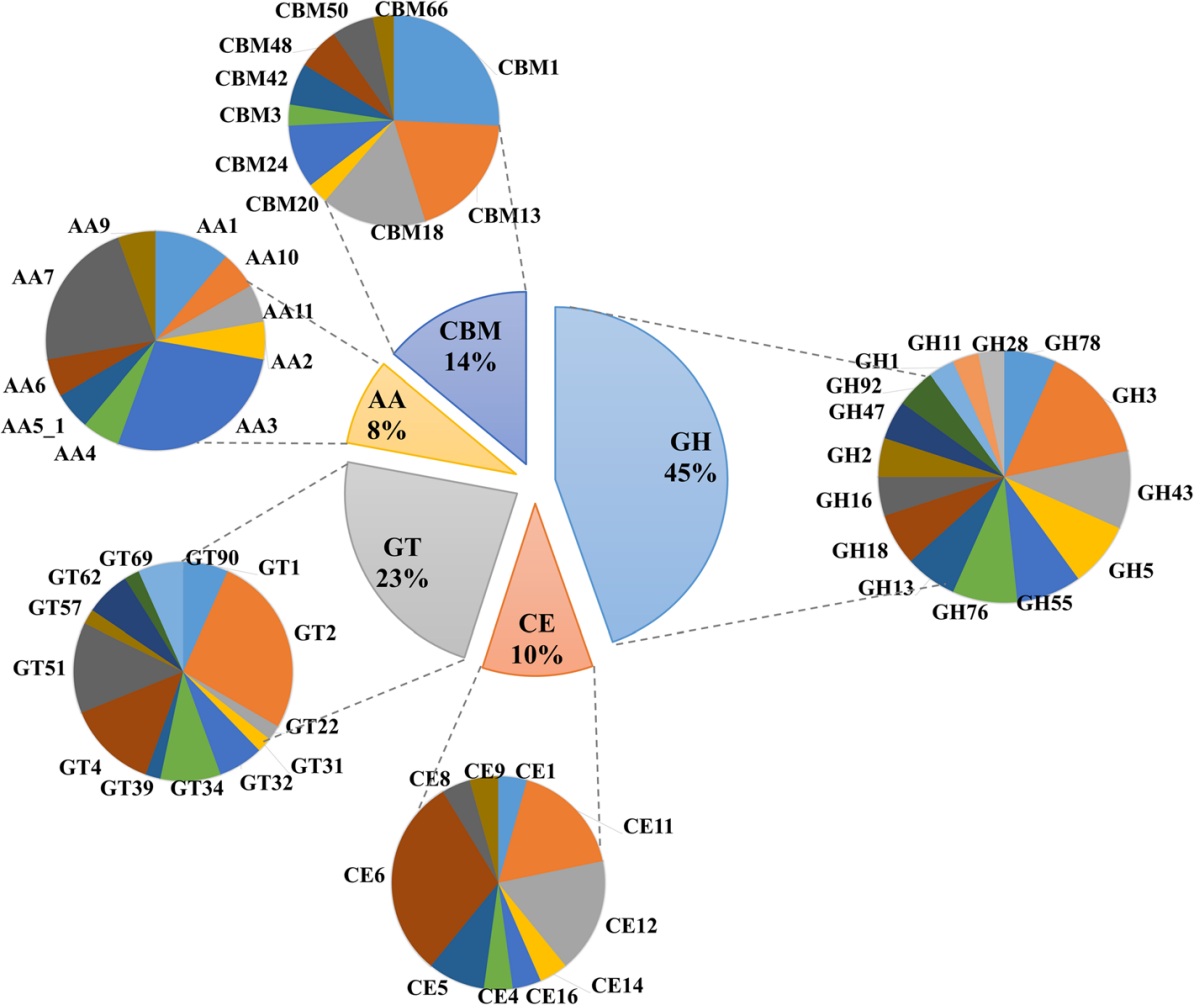
CAZy classification of genes annotated in the genomic regions of *T. harzianum* IOC3844. GH: glycoside hydrolases; GT: glycosyl transferases; PLs: polysaccharide lyases; CEs: carbohydrate esterases; AA: auxiliary activities; CBM: carbohydrate-binding modules

A total of 1676 genes/proteins were predicted (Additional file 3: Supplementary Table S4**)**. Of these, 222 were annotated as CAZymes in ThIOC3844, including 45% annotated as glycoside hydrolases (GHs), 23% annotated as glycosyl transferases (GTs), 10% annotated as carbohydrate esterases (CEs), 8% annotated as auxiliary activities (AAs) and 14% annotated as carbohydrate-binding modules (CBMs) (Fig. 3 and Additional file 5: Supplementary Table S6). The GH class presented with the highest number of families, including GH2 (3 genes), GH7 (1 gene), GH3 (9 genes), GH5 (6 genes), GH12 (1 gene), GH18 (4 genes) and GH62 (1 gene).

### Genomic comparison

For this analysis, we compared the genomic regions of ThIOC3844 against the entire genomes of different strains and species of the genus *Trichoderma*. Genomic comparison of the sequenced regions of ThIOC3844 with two other strains of the same species (*T. harzianum* B97 – ThB97 and *T. harzianum* – T6766) showed a higher similarity to ThB97 (99.25%) than to ThT6766 (91.61%). For the *T. atroviride* IMI206040 genome (TaIMI206040), the similarity to ThIOC3844 was 85.09%. For *T. virens* Gv29–8 (TvGv29–8), the similarity was 86.55%, and for *T. reesei* QM6a (TrQM6a), the similarity was 85.11%.

**Fig. 4:**
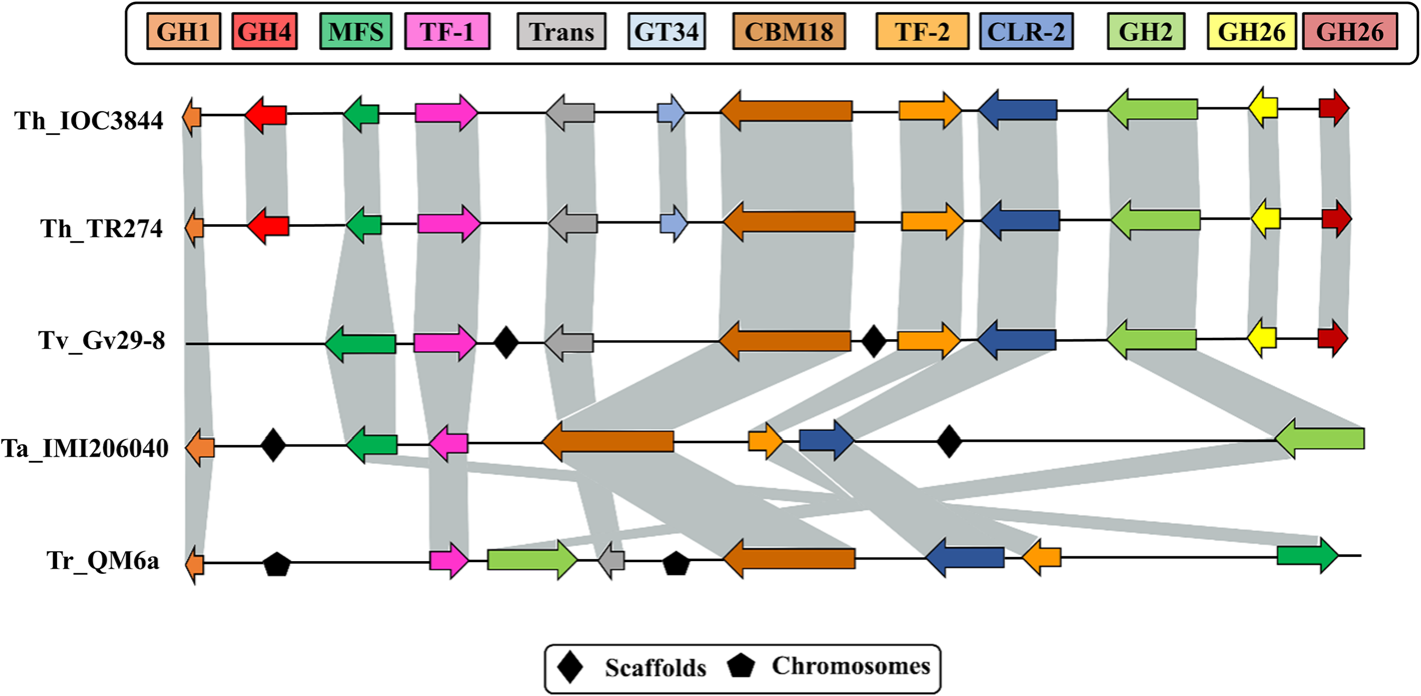
Comparison between the gene clusters of *T. harzianum* IOC3844 and those of other species of the genus *Trichoderma* spp. GH1: glycoside hydrolase 1; GH4: glycoside hydrolase 4; MFS: major facilitator superfamily permease; Trans: putative transporter; TF-1: putative transcription factor 1; GT38: glycosyl transferases 4; CBM18: carbohydrate-binding module 18; TF-2: putative transcription factor 2; CLR2: cellulose regulator 2; GH2: glycoside hydrolase 2; GH26: glycoside hydrolase 26; Th: *T. harzianum*; Tv: *T. virens*; Ta: *T. atroviride*; Tr: *T. reesei*

When we compared syntenic genes between groups of genes, a greater difference between *T. harzianum*, *T. atroviride* and *T. reesei* was observed. The *T. harzianum* TR274 (ThTR274) strain presented the same genomic organization gene profile as ThIOC3844. In TaIMI206040, four genes (GH4, transporter and two GH26) from the cluster were not found; for TvGv29–8, two genes were not found (GH1 and GH4). For *T. reesei* QM6a, three genes (GH4 and two GH26) were not found; in addition, the translocation of genes (MFS x GH2 and TF2 x CLR2) was found. The genes for the TF CLR2, putative TF TF2 and major facilitator superfamily (MFS) permease were maintained in all species analyzed. This result suggests a potential association between the regulation and expression of these genes (Fig. 4).

### Expression determined by RNA-Seq and secreted proteins

All genes predicted in the genomic regions were analyzed according to the expression data by RNA-Seq (under CEL and glucose (GLU) degradation conditions) (Additional file 6: Supplementary Table S7), and secreted proteins were identified by mass spectrometry (LC-MS/MS) (Additional file 7, data obtained in a previous experiments of *T. harzianum* IOC3844 [18]). We found 114 genes with differential expression under CEL degradation conditions compared to GLU degradation conditions; among them, 51 were classified as CAZymes, such as beta-glucosidase of GH1 family (1.8-fold change - FC), LPMOs of the AA9 family (FC 5.0) and a hypothetical protein with CBM1 domain (FC 3.7). In addition, two differentially expressed TFs were identified, CLR2 (FC 1.6) and unidentified transcriptional regulator of zing finger – Zn2Cys6 (FC 2.3). Six transport proteins were also found (iron permease, MFS hexose transporter, siderophore transporter, ammonium permease, sugar transporter and siderophore iron transporter).

Among the genes annotated as CAZymes in ThIOC3844, 31 were found in the secretome of ThIOC3844 under CEL conditions, and the main families were GH3, GH12, CBM1, AA9, GH6/CBM1, GH45/CBM1, GH62 and GH5. In this analysis, we also used the expression levels of the secreted proteins. The gene with the highest transcripts per million (TPM) index (1567.4 TPM) was a cellobiohydrolase (EC 3.2.1.91) of the GH6 family. Our results indicate that genes with low expression levels are also important secreted enzymes (Table 1).

**Table 1:** Proteins identified in genomic and in the *T. harzianum* IOC3844 secretome under cellulose growth conditions

IDs^a^	Protein name	Secretome/UniProt ID	CAZy family	CEL (TPM)	GLU (TPM)
1010	Hypothetical protein	A0A0G0ALT6	GH28	14.2	6.2
1043	Cellulosome enzyme	A0A0G0A296	GH30	35.6	11.5
1054	Glycosyl hydrolase 10	A0A0F9X8A4	GH10	14.8	4.1
1075	Glycosyl hydrolase 64	A0A0F9ZIR5	GH64	824.3	262.4
1095	Glycosyl hydrolase 18	A0A0F9ZHI0	GH18	83.2	47.9
11	Mutanase	A0A0F9XN06	CBM24	2741.6	1452.9
1133	Glycosyl hydrolase 12	A0A0F9Y2E9	GH12	1579.8	308.2
1150	Glycosyl hydrolase 47	A0A0F9WYR7	GH47	83.9	74.6
1217	Beta-mannosidase	A0A0F9ZDV4	GH2	117.9	124.2
126	Glycosyl hydrolase 76	A0A0F9X1Q3	GH76	616.7	375.4
1318	Beta-xylosidase	A0A0G0A408	GH3	172.3	125.2
1439	Alpha-L-arabinofuranosidase B	A0A0G0A4Q2	CBM42	450.4	343.5
1440	Glycosyl hydrolase 3	A0A0F9XRC5	GH3	245.8	107.4
1498	WSC domain-containing	A0A0F9ZXC9	AA5_1	342.5	339.0
44	Beta-1,3-glucanosyltransferase	A0A0F9ZKA8	GH72	2431.7	3210.5
441	Alpha-glucosidase	A0A0G0AG54	GH31	2121.6	1655.2
559	Alpha-1,2-mannosidase	A0A0G0ABI9	GH92	226.9	153.4
666	Glycosyl hydrolase 3	A0A0F9XQT4	GH3	77.2	43.0
667	Hypothetical protein	A0A0G0AME2	CBM1	874.9	142.8
668	Glycosyl hydrolase 61	A0A0F9XMI8	AA9	3109.7	625.1
669	Glycosyl hydrolase 16	A0A0F9XP75	CBM13	16.4	3.7
671	Cytochrome P450 monooxygenase	A0A0G0A4Z5	GT4	1569.5	1595.3
681	Glycosyl hydrolase 11	A0A0F9Y0Y9	GH11/CBM1	4206.8	1316.1
741	Endo-N-acetyl-beta-D-glucosaminidase	A0A0F9ZHA7	GH18	3971.9	2328.0
759	Hypothetical protein	A0A0F9ZJ74	GH20	1184.8	1507.7
813	Catalase peroxidase	A0A0F9X3Z8	AA2	2677.7	2473.4
82	Glycosyl hydrolase 6	A0A0G0AEM7	GH6/CBM1	5843.5	1567.4
842	Hypothetical protein	A0A0F9XY55	GH45/CBM1	41.3	15.3
9	Glycosyl hydrolase 62	A0A0F9X8Z0	GH62	353.9	103.4
913	Isoamyl alcohol oxidase	A0A0F9XC99	AA7	39.3	13.2
918	Hypothetical protein	A0A0F9XG06	GH5_5	870.8	750.9

^a^The annotated genes IDs can be found in Supplementary Table S4

### CLR2 transcription factor

**Fig. 5:**
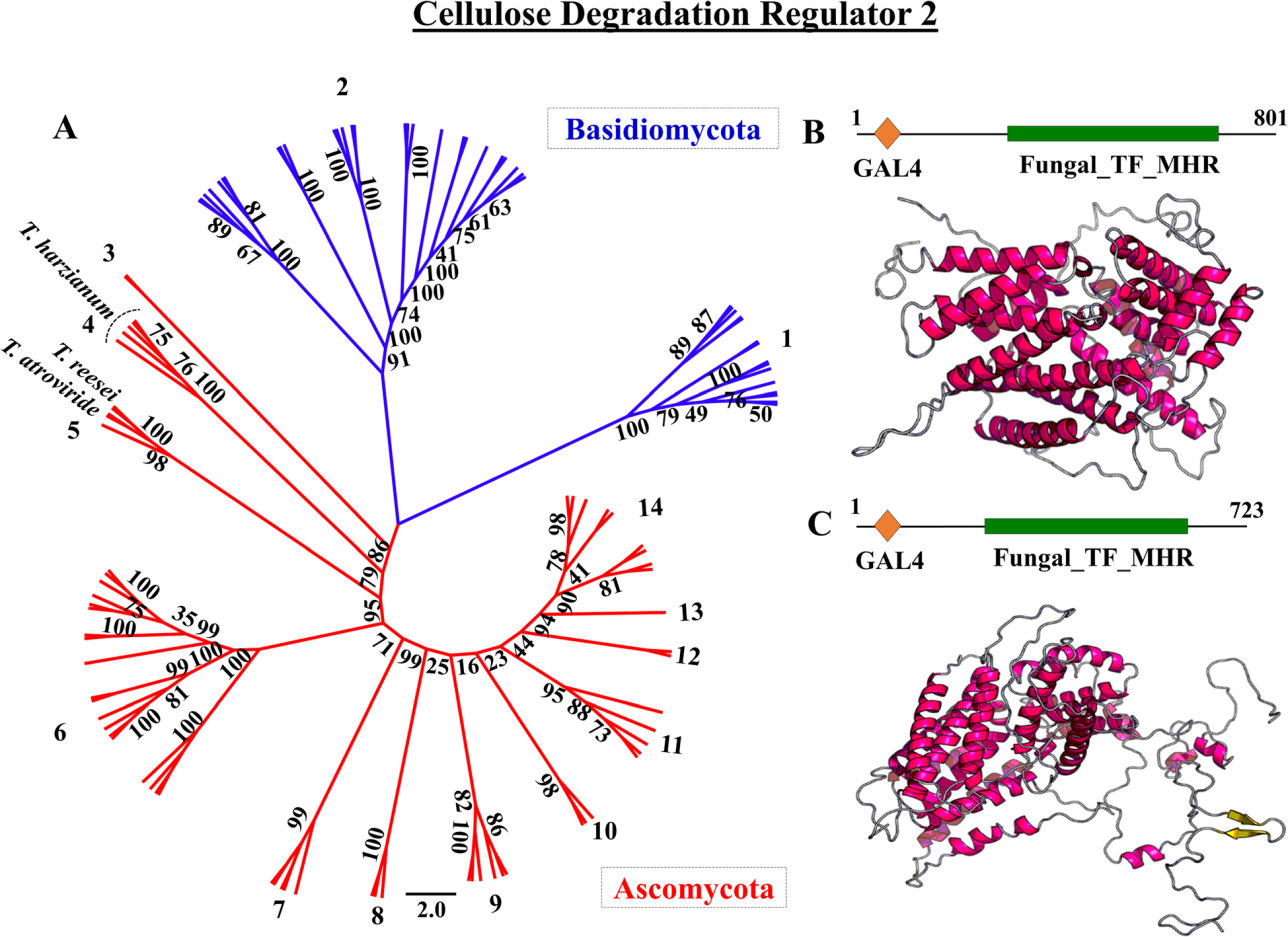
Molecular phylogeny of the CLR2 transcription factor in Ascomycota and Basidiomycota (**a**); in silico protein modeling for CLR2 in *T. harzianum* IOC3844 (**b**) and *T. reesei* QM6a (**c**)

Phylogenetic analysis of the CLR2 factor showed a clear separation of this TF in relation to Basidiomycetes and Ascomycetes (Fig. 5a and Additional file 1: Supplementary Table S8). However, even within these groups, considerable phylogenetic diversity was observed among the species of analyzed fungi with a variety of clades within the same group. Different strains of *T. harzianum* grouped in a single clade with proximity to *T. reesei* and *T. atroviride* species. Our results show a wide range of functional varieties for CLR2, which may indicate different types of performance between species.

Structural modeling analysis of the CLR2 protein of ThIOC3844 was performed using *T. reesei* as a reference. For both proteins, the best template was 6F07 (*Saccharomyces cerevisiae*), with e-values of 4.07e^− 06^ and 6.62e^− 06^ for ThIOC3844 (Fig. 5b) and *T. reesei* (Fig. 5c), respectively. Predictions of 1 and 3 protein domains were made for ThIOC3844 and *T. reesei*, respectively. For ThIOC3844, 59% of the residues were modeled, while *T. reesei* modeled 83% of residues. For ThIOC3844, the secondary structure prediction was 46% H (helix), 0% E (beta-sheet) and 53% C (loop), while solvent access had predictions of 56% E (exposed), 19% M (medium) and 23% B (buried).

**Fig. 6:**
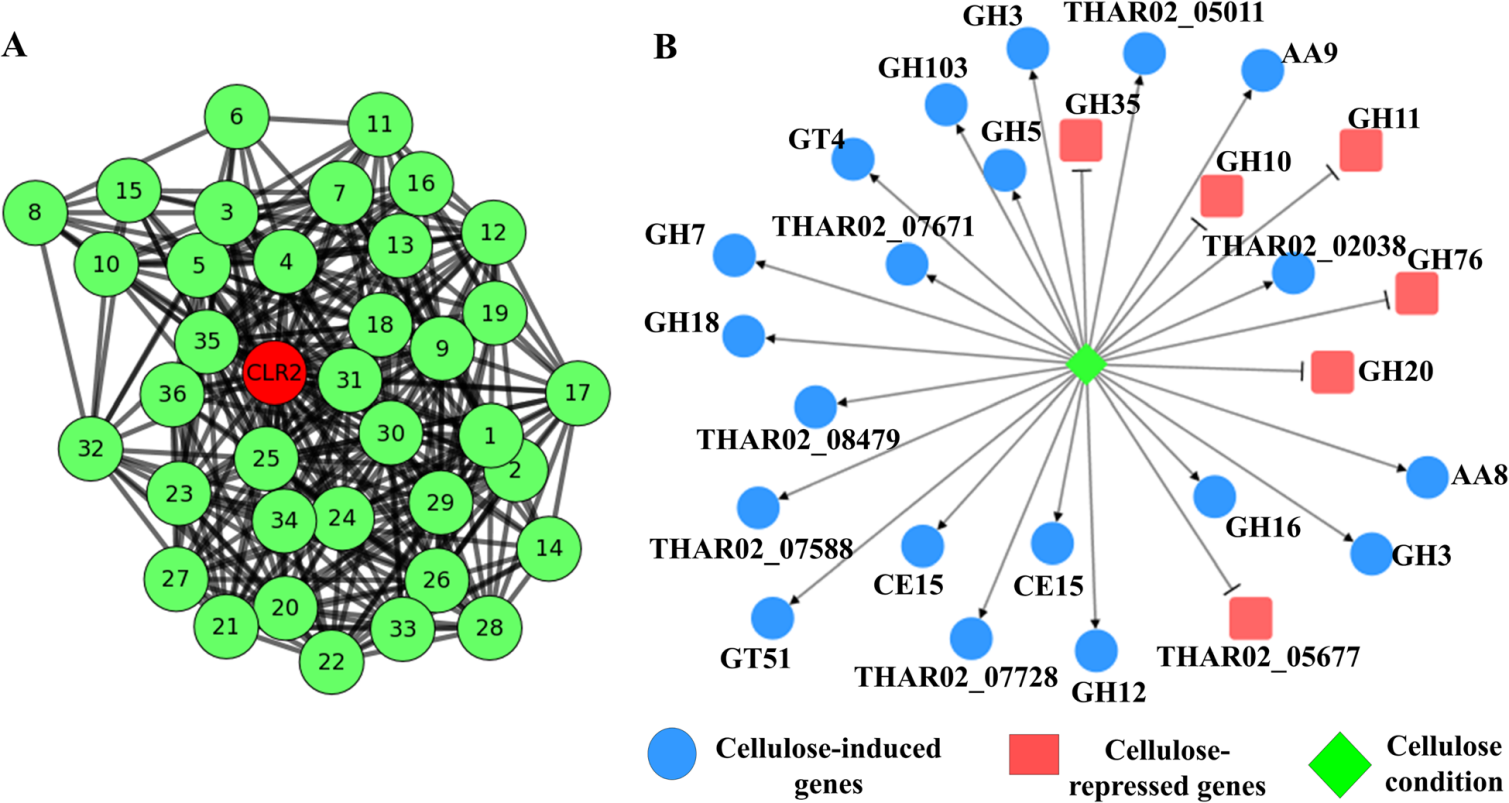
Subnetwork of CLR2 transcription factors and related genes (**a**) and network of induced (blue) and repressed (red) genes under cellulose conditions (**b**). CLR2: cellulose regulator 2; GH: glycoside hydrolases; GT: glycosyl transferases; AA: auxiliary activities

A coregulation network of genes directly related to the CLR2 regulator was constructed, to search for insights about other important proteins in the process of cellulases expression. We identified 36 genes directly linked to CLR2, of which 21 genes were annotated as hypothetical proteins. In addition, genes with known annotations were related to the process of gene expression, including initiation factors, kinases and helicases (Fig. 6a and Additional file 1: Supplementary Table S9).

### Network of induced/repressed genes in cellulose

Using the gene expression data of the secreted proteins, a Bayesian network of induced/repressed genes was constructed based on the CEL growth conditions for *T. harzianum* IOC3844 (Fig. 6b). The major genes that were induced under this condition belong to the GH7 (exoglucanase), GH5 (endo-β-1,4-glucanase), GH3 (β-glucosidase), GH12 (murein transglycosylase), CE15 (CIP2), AA9 (LPMO) and AA8 (hypothetical protein) families. In addition, seven genes that were not classified as CAZymes were also induced under CEL conditions. The families of repressed genes were GH10 (glycoside hydrolase 10 family endo-1,4-β-xylanase), GH11 (glycoside hydrolase 11 family endo-1,4-β-xylanase), GH76 (alcohol dehydrogenase 1), GH20 (β-N-acetylhexosaminidase) and GH35 (glycoside hydrolase 35).

## Discussion

In the present study, an integrative multiomics approach was used to mine CAZyme-rich regions of ThIOC3884. BAC clones were selected, sequenced and used in comparative analyses focusing on the expression profiles via RNA-Seq and the secretome, under different fungal growth conditions. This allowed the discovery of important genes/proteins mechanisms related to plant biomass degradation (Additional file 1: Supplementary Fig. S3).

The vast majority of enzymes that are important for the degradation of plant biomass are already known [28–30]. The current challenge is determining how enzymes are regulated and the genetic mechanism of their activation. Thus, many studies with cellulolytic fungi have focused on TFs, accessory enzymes, transporters and the mechanism by which different types of biomass affects cellulase and hemicellulose regulation [25, 31–33]. Other studies have already shown the potential of *T. harzianum* for the degradation of plant biomass. This is the first study that integrates results from different biotechnological approaches and focuses on the prediction of the most important enzymes and TFs used by *T. harzianum* IOC3844 to degrade CEL.

**Fig. 7:**
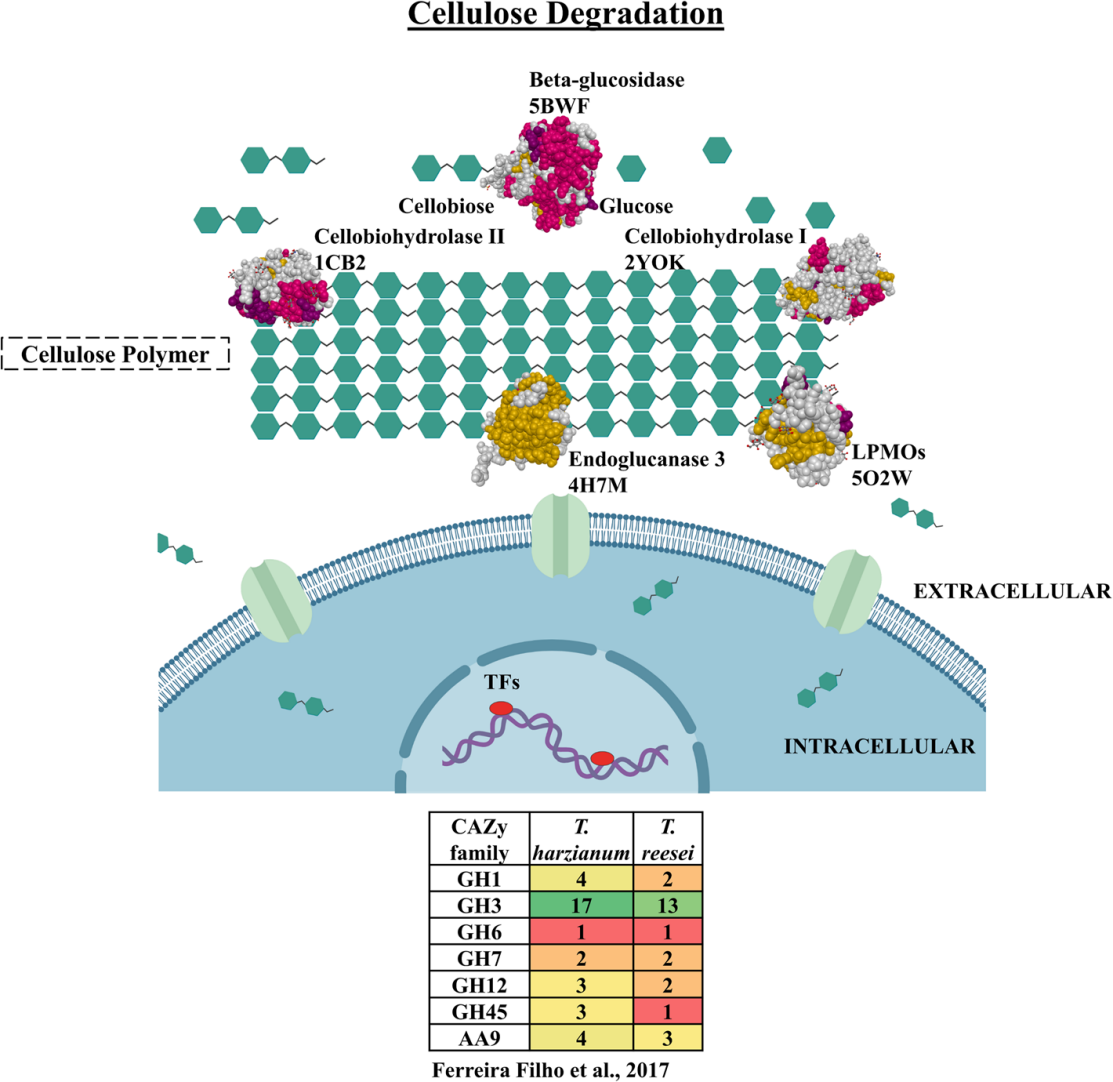
Molecular scheme of the enzymatic model in the degradation of cellulose in *Trichoderma* spp. Enzymes and PDB code: beta-glucosidase (5BWF), cellobiohydrolase I (2YOK), cellobiohydrolase II (1CB2), endoglucanase 3 (4H7M), copper-dependent lytic polysaccharide mono-oxygenases (LPMOs) (5O2W)

The molecular process of CEL degradation is extremely complex and involves hydrolytic enzymes acting on the extracellular medium, carrier proteins and TFs (Fig. 7). For *T. harzianum* and *T. reesei,* the major CAZy families related to CEL degradation were identified in the genome (GH1, GH3, GH6, GH7, GH12, GH45 and AA9) [34], and the three-dimensional structures of cellulases have already been solved. Despite that, many key proteins in the degradation process still not well known, as well as transporters and TFs related to the regulation of related enzymes.

The study of genomic regions is an important tool that provides a global view of the important genes and regulatory regions of a genome [27, 35]. The genomes of few strains of *T. harzianum* are available [36, 37]. A complete genome draft sequenced in 1572 scaffolds is available for *T. harzianum* T6776 [36]; however, little is known about the ThIOC3844 genome, and as this strain has potential for hydrolytic enzymes, more genomic information regarding CAZyme sequences is needed. Our strategy herein aimed to use large genomic regions and integrate these data with other genetic information.

Although the *T. harzianum* T6776 genome is available, it is divided into several small-sized scaffolds [36], which makes impossible in some cases to study gene clusters, regulatory and promoter regions. However, sequencing several regions of the *T. harzianum* IOC3844 genome with single-molecule real-time (SMRT) technology made possible to obtain large scaffolds with reliable assembly. Our sequencing approach allowed to determine the main regions containing important CAZymes involved in the degradation of CEL and hemicellulose, beyond the study of these genes’ clusters.

In the used approach, we chose to select regions of interest instead of sequencing the complete *T. harzianum* IOC3844 genome; this complex approach was only scientifically and economically viable due to previous studies in which we obtained information on the key enzymes in this strain related to the biomass degradation process and optimized an approach for rapid selection and sequencing of large genomic regions for fungi and plants [27, 38]. In this way, we were able to carry out genomic enrichment work quickly and at low cost.

A large number of fungal genomes have already been used as a platform to search for new genes related to the degradation of biomass. It is the case of *T. reesei* QM6a, which has a finalized genome divided into seven chromosomes [39]. Our study results with the genomic regions of ThIOC3844 showed a large number of enzymes classified as CAZymes as well as TFs and transporters in clusters in the genome, important information for future studies on genetic modification of this lineage.

Analyzing the expression levels of determined genes under certain conditions is an important step to understand how transcription is affected in a specific biological condition [12]; however, a direct relationship does not always exist between the gene being highly expressed and the proteins that are important/active in the extracellular medium. Thus, in this work, in addition to studying the genes most expressed in the genomic regions, we also searched for those with a confirmed presence in the fungal secretome under CEL degradation conditions. Our results described the key CAZy families to the degradation of CEL, with high levels of expression and a positive presence as secreted proteins.

Genomic comparison is a powerful tool for understanding differences and evolutionary dynamics among related species [40–42]. Our data show a high similarity among different strains of *T. harzianum* (IOC3844, B97 and T6776), which indicates that differences in enzyme production and efficiency may be related more to gene regulation mechanisms than to differences in the sequence itself. In addition, synteny analysis showed a greater difference in relation to the *T. reesei* genome, which can be explained by the loss of genes and genomic modifications carried out in *T.reesei* lineages that potentially increases the production of enzymes related to plant biomass degradation [13, 43].

Despite the fact that genomic sequences of different *T. harzianum* strains are highly similar, studies from our group showed that stains differences can be observed in terms of regulation of CEL degradation-related genes and in the enzyme activity profiles [17, 18]. Therefore, punctual genomic differences between *T. harzianum* strains (such as SNPs in introns and gene regions) can play an important role in the efficiency of strains.

The CLR2 TF was described as an important regulator of cellulase expression on *Neurospora crassa* [25]; however, CLR2 functional role in fungi of the genus *Trichoderma*, including *T. reesei*, remains unclear [15, 44]. In the ThIOC3844 genome, we found a cluster with the CLR2 TF in association with other putative TFs, CAZymes, transporters and MFS permease. The same behavior was found for the *T. reesei* CLR2 TF, which has physical proximity and is coexpressed with a sugar transporter [32, 45]. These results indicate a mechanism for joint regulation and expression of this TF with transporters related to biomass degradation. RNA-Seq data showed differential expression of CLR2 under CEL conditions. In this way, we analyzed the coregulation network of the CLR2 regulator. The present study illuminates unclear areas of the genomic organization, expression, and putative regulation of CLR2 in *T. harzianum*.

Coregulation networks provide insights into how genes correlate and interact with each other [46, 47]. We identified 36 genes directly associated with the CLR2 regulatory factor; these genes may be important in the regulatory process of this factor, which is linked to the expression of cellulases in other filamentous fungi. Techniques such as gene knockout can further validate the functional or synergistic importance of these genes with key TFs for the expression of genes related to the degradation of plant biomass.

## Conclusions

Our results present an innovative approach of using different types of omics data to search for new important genes and genetic regulation mechanisms during the process of CEL degradation. We found several TFs, accessory enzymes, and transporters in the genomic regions of ThIOC3844 that may be important for the expression/secretion of CAZyme genes. Among these, CLR2, CIP2 and LPMOs are promising candidates for further study. Our results indicate that the CRL2 regulator matches all the requirements for involvement in CEL degradation by *T. harzianum*. In addition, the use of coregulation networks makes it possible to understand the relationship between genes and to find new targets for biochemical characterization. The results allowed the identification of important genetic regions, key genes and functional proteins, and this information can be used for the development and improvement of enzymatic hydrolysis technology for the bioethanol industry.

## Methods

### T. Harzianum strain and genomic resources

*T. harzianum* IOC3844 (ThIOC3844) was obtained from Institute Oswaldo Cruz (IOC, Rio de Janeiro, Brazil) and deposited on the Brazilian Collection of Environment and Industrial Microorganisms (CBMAI, Campinas, Brazil). A library of BACs consisting of 5760 clones previously constructed for this fungal strain [27] was used to search for genomic regions. The genomic sequences of *T. harzianum* T6776 (PRJNA252551), *T. reesei* QM6a (PRJNA325840), *T. atroviride* IMI206040 (PRJNA19867) and *T. virens* Gv29–8 (PRJNA19983) were used for comparison with ThIOC3844.

### BAC library screening for gene selection in T. harzianum IOC3844

We designed primers for 62 target CAZyme genes (Additional file 2: Supplementary Table S1) using transcriptome data [4] to search for positive BAC clones that contain genes previously selected. A pool of clones from each plate that made up the BAC library was used for selection (the complete BAC library consisted of fifteen 384-well plates), and pools with clones of all columns of the plates (24 columns on each plate) were also used. In this way, three selections were made to find the positive clone, searches in the plate pool, in the column pool and in the positive column, to obtain the coordinates of the positive clones. The plate and column pools were amplified using the Illustra GenomiPhi HY DNA Amplification Kit (GE Healthcare Life Sciences, UK) according to the manufacturer’s instructions. The screening reactions for the search for positive clones were performed via PCR using the CFX384 Touch Real-Time PCR Detection System (Bio-Rad).

### Single-molecule real-time (SMRT) sequencing and assembly

Libraries for sequencing were prepared according to the Pacific Biosciences (PacBio) protocol, and sequencing was performed at the Arizona Genomics Institute (AGI; Tucson, USA) using a SMRT DNA sequencing system available from PacBio (PacBio RSII platform). The sequences were deposited into the NCBI SRA databank under bioproject number PRJNA647392. De novo assembly was performed with the PacBio Corrected Reads (PBcR) pipeline implemented as part of Wgs-assembler v8.3rc2 [48] and Celera Assembler [49]. The contigs obtained with the assemblers were subjected to error correction with pbalign (v0.2). The PacBio reads were aligned using the BLASR algorithm [50], and assembly polishing was performed with the Quiver tool (accession numbers MK861589-MK861650 and Additional file 1: Supplementary Table S2 and S3) [51].

### Gene prediction and functional annotation

The FGENESH tool was used for initial gene prediction analysis [52], followed by manual correction with the *T. harzianum* T6776 and *T. reesei* QM6a gene models. Annotations of the ontologies were performed with Blast2GO [53]. InterPro protein domains were predicted using InterProScan (http://www.ebi.ac.uk/interpro/) [54]. Information derived from the CAZy database was downloaded for each CAZyme family (www.cazy.org). The protein sequences of *T. harzianum* IOC3844 were used as queries in basic local alignment search tool (BLASTp) searches against the locally built CAZyme BLAST database. Only BLAST matches showing an e-value less than e-10, identity greater than 30% (the identity value allows capture of the similarity between proteins that have conserved functional domains, as is the case for proteins with CBM domains), and queries covering more than 70% of the sequence length were retained and classified as GHs, GTs, polysaccharide lyases (PLs), CEs, CBMs or AAs according to the CAZyme catalytic activities.

### Genomic comparison in Trichoderma spp.

The genomes of *Trichoderma* (*T. harzianum*, *T. virens*, *T. atroviride* and *T. reesei*) were compared with that of *T. harzianum* IOC3844 using global alignment through Nucmer (−maxmatch), which is part of the software package MUMmmer 3.23 [55]. Delta-filter (−q), show-coords (−rcl), and DNADIFF (standard parameters) were used for filtering, obtaining the mapping coordinates and generating the statistical report in the alignment, respectively. SimpleSynteny software (https://www.dveltri.com/simplesynteny/) [56] was used to compare a cluster of 12 genes (in physical proximity to the CLR2 TF) among different species of *Trichoderma* spp.

### Phylogenetic analysis and structural modeling of CLR2

The CLR2 sequences of ThIOC3844, *T. reesei* QM6a, *T. atroviride*, *T. virens* and other species of fungi were used as the basis for constructing the phylogenetic trees. These fungi were divided into Ascomycetes and Basidiomycetes. The sequences were aligned using ClustalW [57] and analyzed with Molecular Evolutionary Genetics Analysis (MEGA) software v7.0 (https://www.megasoftware.net/) [58]. The phylogenetic analyses were performed in MEGA7 using the maximum likelihood (ML) [59] method of inference based on the Jones-Taylor-Thornton (JTT) matrix-based model and 1000 bootstrap replicates [60]. Pairwise deletion was employed to address alignment gaps and missing data. The trees were visualized and edited using the FigTree program (http://tree.bio.ed.ac.uk/software/figtree/). In silico modeling of the CLR2 domain was performed using RaptorX protein structure prediction software (http://raptorx.uchicago.edu/) [61].

### RNA-Seq and secreted protein analysis

The ThIOC3844 expression levels were analyzed using RNA-Seq data (PRJNA336221) obtained from a previous study in which the transcripts were obtained following growth of the fungus on two different carbon sources, CEL and GLU [18]. The reads from the RNA-Seq library were mapped against those of the ThIOC3844 genes using the CLC Genomics Workbench (QIAGEN, Aarhus, Denmark) [62]. The expression values are expressed in reads per kilobase of exon model per million mapped reads (RPKM), and the normalized value for each sample was calculated in TPM. For the analysis of differential expression, the following parameters were used: fold change (FC) greater than or equal to 1.5 and a *p*-value lower than 0.05. Secreted proteins were analyzed using a Blastp search of the annotated proteins in ThIOC3844 against a local protein database (Additional file 7**)** generated by a previous study that determined the proteins secreted by the fungus under CEL and GLU conditions using a liquid chromatography tandem mass spectrometry (LC-MS/MS) technique [18].

### Gene regulatory network

The gene regulatory networks were assembled from the reference mapped RNA-Seq data using each set of biological triplicates for the CEL and GLU conditions [18]. The interaction between the genes was obtained by calculating Pearson’s correlation for each pair of genes. The induction and repression networks were constructed based on the expression data of a set of genes that were identified in the secretome of the CEL growth condition by the Bayesian inference method [63]. If the secreted protein was present in the condition, it was assigned a value of one. If the secreted protein was absent, it was assigned a value of zero. The treatment conditions were considered regulators of the network to detect the direct relationships between the conditions and the genes. Thus, the Bayesian network represents the relationships among the conditions, gene expression, and secreted proteins. Cytoscape software v 3.4.042 [64] (https://cytoscape.org/) was used for data analysis and construction of the CLR2 subnetwork.

## Supplementary information

**Additional file 1 Fig. S1.** Screening genes of interest in the genomic library of *T. harzianum* IOC3844 by qPCR (a); read size sequenced using PacBio technology (b); genes clustered in a genomic region of *T. harzianum* (c). **Fig. S2.** Distribution of the main GO terms of the annotated genes in *T. harzianum* IOC3844. **Fig. S3.** Pipeline approach for the analyses used in this study of genes and genomes in *T. harzianum*. **Supplementary Table S2.** Assembly parameters of a set of sequenced genomic regions using PacBio technology. **Supplementary Table S3.** Comparison of genomic data among different species of *Trichoderma* spp. **Supplementary Table S8.** Description of the species used for the phylogenetic analysis of the transcription factor CLR2. **Supplementary Table S9.** Description of the genes found in the coregulation networks.

**Additional file 2 Supplementary Table S1.** Description of the genomic regions sequenced in *T. harzianum* IOC3844.

**Additional file 3 Supplementary Table S4.** Annotation of all genes predicted in *T. harzianum* IOC3844.

**Additional file 4 Supplementary Table S5.** Description of the EC codes for *T. harzianum* IOC3844 genes.

**Additional file 5 Supplementary Table S6.** Description of the CAZyme genes in *T. harzianum* IOC3844.

**Additional file 6 Supplementary Table S7.** Expression levels of the genes annotated in *T. harzianum* IOC3844 by means of RNA-Seq.

**Additional file 7 **FASTA sequences of proteins secreted in *T. harzianum* IOC3844 under cellulose conditions developed in the work of Horta et al., 2018.

## Data Availability

The raw data of the genomic regions (PacBio reads) can be found by the accession number PRJNA647392. The raw data of the RNA-Seq (Illumina reads) can be found by the accession number PRJNA336221. Assemblies and annotations (Additional file 3: Supplementary Table S4) from the genomic regions were submitted to GenBank (https://www.ncbi.nlm.nih.gov/genbank/) under the accession numbers MK861589-MK861650 (Additional file 2: Supplementary Table S1 and Additional file 3: Supplementary Table S4).

## Abbreviations

AA: Auxiliary activities
B: Buried
BAC: Bacterial artificial chromosome
BLAST: Basic local alignment search tool
bp: Base pair
BRENDA: Braunschweig enzyme database
C: Loop
CAZymes: Carbohydrate-active enzymes
CBMAI: Brazilian collection of environment and industrial microorganisms
CBM: Carbohydrate-binding module
CE: Carbohydrate esterases
CEL: Cellulose
CIP1: Cellulose-induced protein 1
CIP2: Cellulose-induced protein 2
CLR2: Cellulose degradation regulator 2
DNA: Deoxyribonucleic acid
E: Beta-sheet
EC: Enzyme commission number
Ex: Exposed
FC: Fold change
GH: Glycoside hydrolases
GLU: Glucose
GO: Gene ontology
GT: Glycosyltransferases
H: Helix
JTT: Jones-Taylor-Thornton
kb: Kilobases
LPMO: Lytic polysaccharide monooxygenase
M: Medium
Mb: Megabase
MEGA: Molecular evolutionary genetics analysis
MFS: Major facilitator superfamily permease
ML: Maximum likelihood
PacBio: Pacific biosciences
PBcR: PacBio corrected reads
PCR: Polymerase chain reaction
PL: Polysaccharide lyases
RNA: Ribonucleic acid
RNA-Seq: RNA sequencing
RPKM: Reads per kilobase of exon model per million mapped reads
SMRT: Single-molecule real-time
TaIMI206040: *T. atroviride* IMI206040
TF: Transcription factor
ThB97: *T. harzianum* B97
ThIOC3844: *Trichoderma harzianum* IOC-3844
ThTR274: *T. harzianum* TR274
Th6766: *T. harzianum*
TPM: Transcripts per million
TrQM6a: *T. reesei* QM6a
TvGv29–8: *T. virens* Gv29–8
