# Supplementary material for: “Integrative genomic analysis of the bioprospection of regulators and accessory enzymes associated with cellulose degradation in a filamentous fungus (Trichoderma harzianum)”
Source: BMC Genomics. 2020 Nov 2;21:757. doi: 10.1186/s12864-020-07158-w (PMC7607812; doi:10.1186/s12864-020-07158-w)
Supplement: Supplementary file 1 — Additional file 1 Fig. S1. Screening genes of interest in the genomic library of T. harzianum IOC3844 by qPCR (a); read size sequenced using PacBio technology (b); genes clustered in a genomic region of T. harzianum (c). Fig. S2. Distribution of the main GO terms of the annotated genes in T. harzianum IOC3844. Fig. S3. Pipeline approach for the analyses used in this study of genes and genomes in T. harzianum. Supplementary Table S2. Assembly parameters of a set of sequenced genomic regions using PacBio technology. Supplementary Table S3. Comparison of genomic data among different species of Trichoderma spp. Supplementary Table S8. Description of the species used for the phylogenetic analysis of the transcription factor CLR2. Supplementary Table S9. Description of the genes found in the coregulation networks. [file 12864_2020_7158_MOESM1_ESM.pdf]

"Integrative Genomic Analysis for the Bioprospection of Regulators and Accessory Enzymes Associated with Cellulose Degradation in a Filamentous Fungus (*Trichoderma harzianum*)"

Jaire A. Ferreira Filho[1,2], Maria Augusta C. Horta[1,3], Clelton A. dos Santos[1], Deborah A. Almeida[1,2], Natália F. Murad[1], Juliano S. Mendes[1], Danilo A. Sforça[1], Claudio Benício C. Silva[1], Aline Crucello[1], Anete P. de Souza[1,4,*]

[1]Center for Molecular Biology and Genetic Engineering (CBMEG), University of Campinas (UNICAMP), Campinas, SP, Brazil

[2]Graduate Program in Genetics and Molecular Biology, Institute of Biology, UNICAMP, Campinas, SP, Brazil

[3]Holzforshung München, TUM School of Life Sciences Weihenstephan, Technische Universität München, 85354 Freising, Germany

[4]Department of Plant Biology, Institute of Biology, UNICAMP, Campinas, SP, Brazil

Supplementary data

This PDF file includes:

Figs S1 to S3

Tables S2, S3, S8 and S9

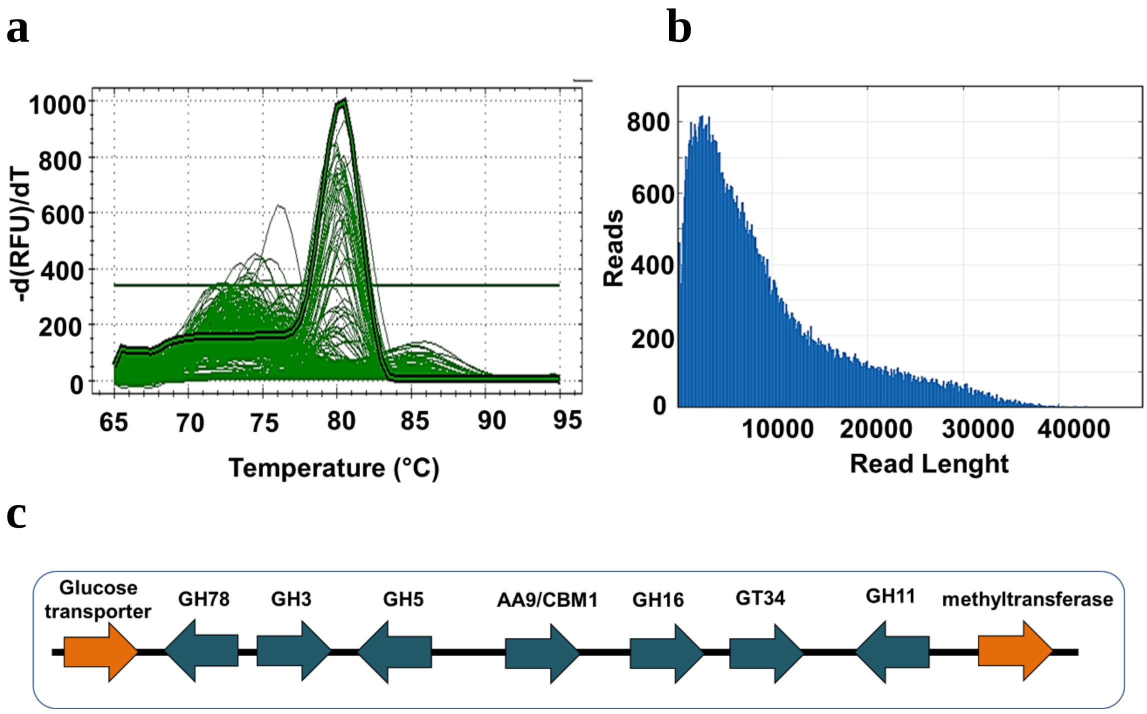

Fig. S1. Screening genes of interest in the genomic library of *T. harzianum* IOC3844 by qPCR (a); reads size sequenced using PACBio technology (b); genes cluster in a genomic region of *T. harzianum* (c).

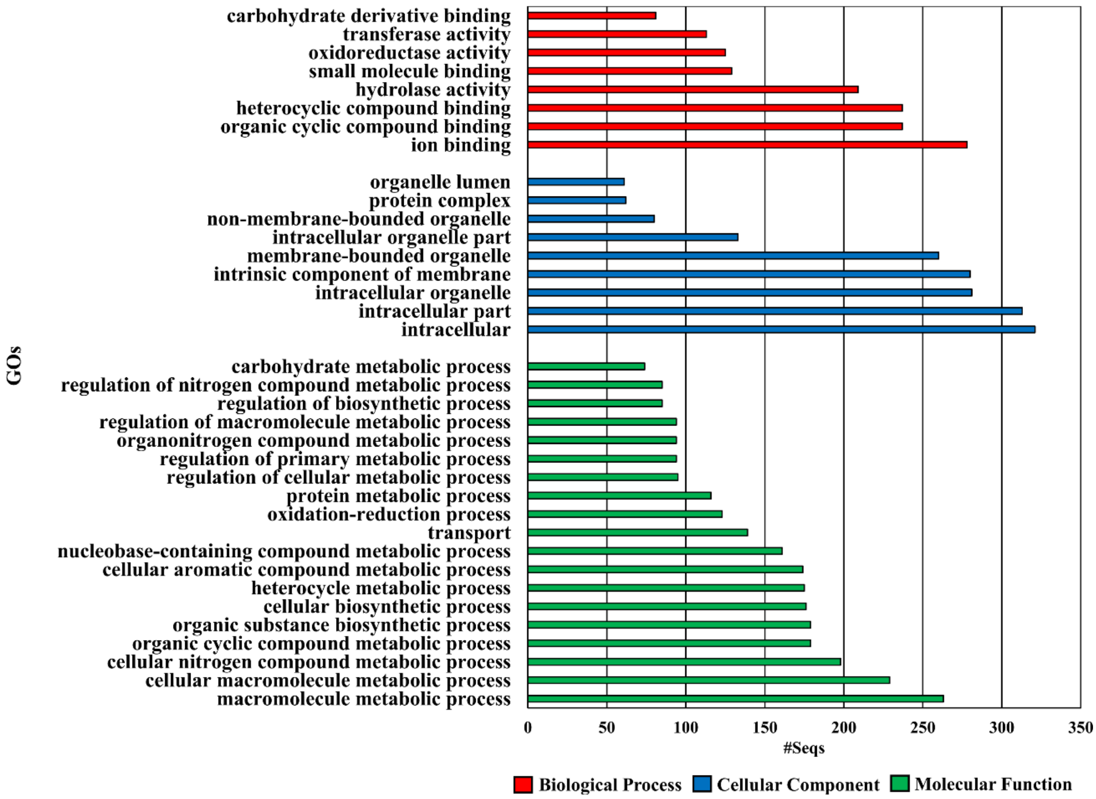

Fig. S2. Distribution of the main GO terms of the annotated genes in *T. harzianum* IOC3844.

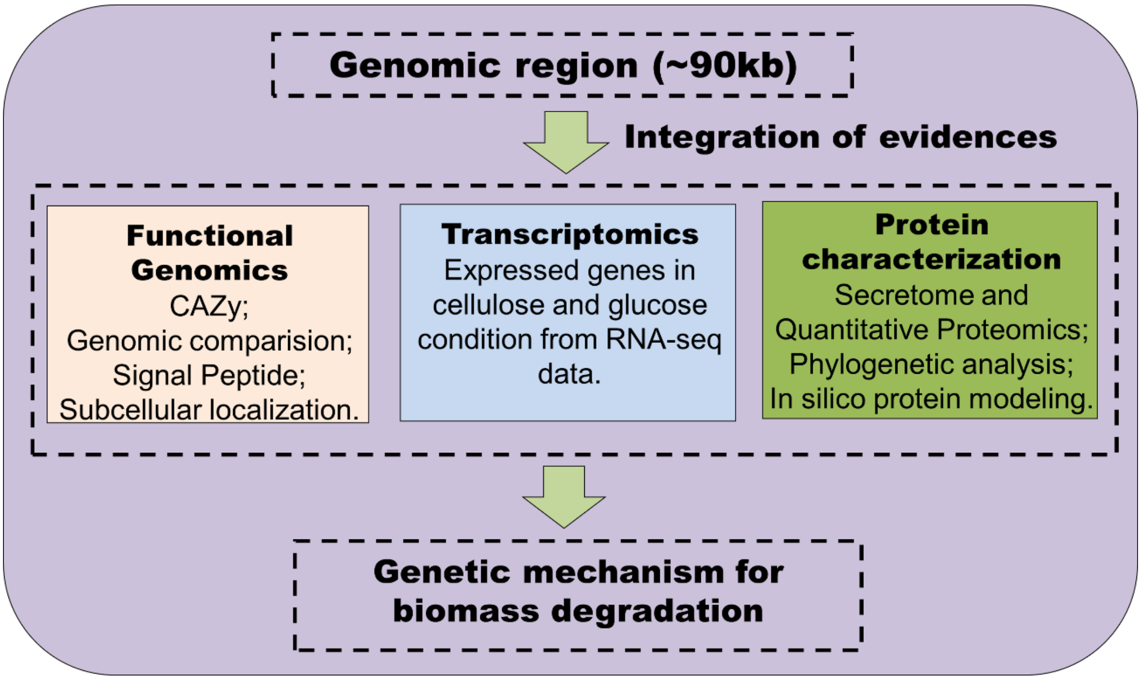

Fig. S3. Pipeline approach for the analyzes used in this work of genes and genomic study in *T. harzianum*.

Supplementary Table S2. Assembly parameters of a set of sequenced genomic region using PACBio technology.

Parameters	Results
Polymerase Read Bases	1151876133
Seed Bases	150009116
Pre-Assembly Yield	0.296
Pre-Assembly Reads Length	5528
Length Cutoff	12815
Pre-Assembly bases	44419992
Pre-Assembly Reads	8035
Pre-Assembly N50	8199
Mean Read Score	0.667
Number of Reads	100732
Mean Read Length	11493
Number of bases	1157715225
N50 Read Length	17608
Polymerase Read Quality	0.849

Supplementary Table S3. Comparison of genomic data among different species of *Trichoderma* spp.

**Organism/Name**	**Strain**	**Genome type**	**Size (Mb)**	**GC%**	**Scaffolds**	**CAZymes**
T. harzianum	IOC3844	BACs	5.00	49.65	63	222
T. harzianum	T6776	Draft	40.98	48.50	1572	430
T. reesei	QM6a	Complete genome	33.39	52.80	7	327
T. atroviride	IMI 206040	Draft	36.14	49.70	29	422
T. virens	Gv29-8	Draft	39.00	49.20	93	441

Supplementary Table S8. Description of the species used for the phylogenetic analysis of the transcription factor CLR2.

Group	Sequence ID	Species
1	XP_012182108.1	*Fibroporia radiculosa*
	KZT72632.1	*Daedalea quercina* L-15889
	PCH41399.1	*Wolfiporia cocos* MD-104 SS10
	XP_024336022.1	*Postia placenta* MAD-698-R-SB12
	OCH94255.1	*Obba rivulosa*
	PSR73669.1	*Phlebia centrifuga*
	XP_007392271.1	*Phanerochaete carnosa* HHB-10118
	XP_008035817.0	*Trametes versicolor* FP-101664 SS1
	XP_007364283.1	*Dichomitus squalens* LYAD-421
	OJA15282.1	*Rhizopogon vesiculosus*
	OAX40653.1	*Rhizopogon vinicolor* AM-OR11-026
	KIJ70318.1	*Hydnomerulius pinastri* MD-312
	KIK30790.1	*Pisolithus microcarpus* 441
	KIO13022.1	*Pisolithus tinctorius* Marx 270
2	OXG14340.1	*Cryptococcus neoformans* var. grubii Tu2591
	OWZ51650.1	*Cryptococcus neoformans* var. grubii 125.91
	OXG77158.1	*Cryptococcus neoformans* var. grubii Br795
	OWZ35064.1	*Cryptococcus neoformans* var. grubii AD1-83a
	XP_012051901.1	*Cryptococcus neoformans* var. grubii H99
	OWT36835.1	*Cryptococcus neoformans* var. grubii Bt1
	OXG13205.1	*Cryptococcus neoformans* var. grubii Tu401-1
	OWZ76252.1	*Cryptococcus neoformans* var. grubii Bt85
	OXM75578.1	*Cryptococcus neoformans* var. grubii Bt63
	XP_018260593.1	*Kwoniella dejecticola* CBS 10117
	XP_018999125.1	*Kwoniella mangroviensis* CBS 8507
	XP_019045940.1	*Kwoniella bestiolae* CBS 10118
	XP_019014217.1	*Kwoniella pini* CBS 10737
	XP_018266380.1	*Kwoniella dejecticola* CBS 10117
	OCF60695.1	*Kwoniella mangroviensis* CBS 10435
	OCF74556.1	*Kwoniella mangroviensis* CBS 8886
	XP_019001153.1	*Kwoniella mangroviensis* CBS 8507
	XP_021869321.1	*Kockovaella imperatae*
	XP_019045848.1	*Kwoniella bestiolae* CBS 10118
	XP_018260036.1	*Kwoniella dejecticola* CBS 10117
	XP_003196556.1	*Cryptococcus gattii* WM276

2	OWZ29927.1	*Cryptococcus neoformans* var. grubii c45
	XP_019010711.1	*Kwoniella pini* CBS 10737
	POY75593.1	*Rhodotorula taiwanensis*
	XP_018280147.1	*Cutaneotrichosporon oleaginosum*
	EKD01798.1	*Trichosporon asahii* var. asahii CBS 8904
	XP_014181811.1	*Trichosporon asahii* var. asahii CBS 2479
3	CDM38126	*Penicillium roqueforti* FM164
	XP_660973.1	*Aspergillus nidulans* FGSC A4
4	PKK47514.1	*Trichoderma harzianum* CL102_9848
	Th_IOC3844	*Trichoderma harzianum* IOC3844
	XP_024778108.1	*Trichoderma harzianum* CBS 226.95
	KKP03054.1	*Trichoderma harzianum*
	XP_013955330.1	*Trichoderma virens* Gv29-8
5	ETS03553.1	*Trichoderma reesei* RUT C-30
	XP_006964036.1	*Trichoderma reesei* QM6a
	XP_013941191.1	*Trichoderma atroviride* IMI 206040
	XP_024764890.1	*Trichoderma asperellum* CBS 433.97
	KOS18048.1	*Escovopsis weberi*
6	CZT51594.1	*Rhynchosporium secalis*
	CZT06835.1	*Rhynchosporium agropyri*
	CZT07141.1	*Rhynchosporium commune*
	PMD16076.1	*Pezoloma ericae*
	PMD30568.1	*Hyaloscypha variabilis* F
	XP_024739234.1	*Meliniomyces bicolor* E
	XP_018061285.1	*Phialocephala scopiformis*
	CZR63207.1	*Phialocephala subalpina*
	XP_007288749.1	*Marssonina brunnea*
	PQE16296.1	*Rutstroemia* sp. NJR-2017a WRK4
	PQE03599.1	*Rutstroemia* sp. NJR-2017a BBW
	ESZ95397.1	*Sclerotinia borealis* F-4128
	EMR81895.1	*Botrytis cinerea* BcDW1
	XP_024552100.1	*Botrytis cinerea* B05.10
	CCD51977.1	*Botrytis cinerea* T4
	KFX95591.1	*Pseudogymnoascus* sp. VKM F-3557
	OBT56849.1	*Pseudogymnoascus* sp. 24MN13
	KFZ12187.1	*Pseudogymnoascus* sp. VKM F4519 (FW-2642)
	XP_018129406.1	*Pseudogymnoascus verrucosus*
7	KUI68282.1	*Valsa mali*

7	KUI55118.1	*Valsa mali* var. pyri
	POS76166.1	*Diaporthe helianthi*
	KKY36004.1	*Diaporthe ampelina*
	PSS02243.1	*Coniella lustricola*
8	XP_016589835.1	*Sporothrix schenckii* 1099-18
	KIH94787.1	*Sporothrix brasiliensis* 5110
	EPE08882.1	*Ophiostoma piceae* UAMH 11346
9	XP_009849767.1	*Neurospora tetrasperma* FGSC 2508
	XP_962712.2	*Neurospora crassa* OR74A
	XP_003347695.1	*Sordaria macrospora* k-hell
	XP_006693821.1	*Chaetomium thermophilum*
	KXX83256.1	*Madurella mycetomatis*
	XP_003660436.1	*Thermothelomyces thermophila*
10	KLU85969.1	*Magnaporthiopsis poae* ATCC 64411
	XP_009224916.1	*Gaeumannomyces tritici* R3-111a-1
	XP_003714853.1	*Magnaporthe oryzae* 70-15
11	OTA63138.1	*Hypoxylon* sp. EC38
	OTA97472.1	*Hypoxylon* sp. CO27-5
	OTB08885.1	*Hypoxylon* sp. CI-4A
	OTB14150.1	*Daldinia* sp. EC12
	KXJ95218.1	*Microdochium bolleyi*
12	ELA25990.1	*Colletotrichum fructicola* Nara gc5
	ENH84300.1	*Colletotrichum orbiculare* MAFF 240422
13	OLN97553.1	*Colletotrichum chlorophyti*
14	KXH28512.1	*Colletotrichum nymphaeae* SA-01
	KXH27412.1	*Colletotrichum simmondsii*
	XP_022478568.1	*Colletotrichum orchidophilum*
	KXH59612.1	*Colletotrichum salicis*
	XP_008097168.1	*Colletotrichum graminicola* M1.001
	KDN69204.1	*Colletotrichum sublineola*
	KZL75422.1	*Colletotrichum tofieldiae*
	OHW92794.1	*Colletotrichum incanum*
	KZL81134.1	*Colletotrichum incanum*

Supplementary Table S9. Description of the genes found in the coregulation networks.

Network ID	Protein ID	Protein name
1	KKP06817.1	Hypotetical protein
2	KKP05702.1	Hypotetical protein
3	KKP00617.1	Hypotetical protein
4	KKP03936.1	Kinase DC2
5	KKP07737.1	Translation initiation factor 3 subunit k
6	KKP01671.1	Hypotetical protein
7	KKO98378.1	Translation initiation factor subunit 1
8	KKP03537.1	Murein transglycosylase
9	KKO96961.1	Hypotetical protein
10	KKP00812.1	Hypotetical protein
11	KKP06268.1	Hypotetical protein
12	KKP01476.1	Peptidyl-prolyl-cis-trans isomerase sspl
13	KKO97887.1	Hypotetical protein
14	KKO99717.1	Hypotetical protein
15	KKP00810.1	Hypotetical protein
16	KKP07167.1	Hypotetical protein
17	KKP02416.1	Hypotetical protein
18	KKO98063.1	Vacuolar ATP synthase subunit D
19	KKP03148.1	Hypotetical protein
20	KKP07719.1	Hypotetical protein
21	KKO97889.1	Hypotetical protein
22	KKP00888.1	ATP-dependent RNA helicase DED1
23	KKP02267.1	Ubiquitin-protein ligase E3 C
24	KKP02972.1	Hypotetical protein
25	KKO98726.1	DUF718
26	KKP02131.1	Hypotetical protein
27	KKO96618.1	Hypotetical protein
28	KKO98708.1	Hypotetical protein
29	KKP02590.1	Proteasome subunit alpha type-2
30	KKO99933.1	Hypotetical protein
31	KKP07598.1	Undercaprenyl diphosphate synthase
32	KKP00804.1	14-3-3 family protein
33	KKP02280.1	ATP-dependent RNA helicase SUB2
34	KKP03054.1	Cellulose Degradation regulator 2 – CLR2
35	KKO97002.1	Hydroxyacylglutathione hydrolase
36	KKP01112.1	Hypotetical protein
